# Treatment of Amenorrhœa

**Published:** 1891-09

**Authors:** 


TREATMENT OF AMENORRHŒA.—

| | | |
|---|---|---|
| R. | Bichloride of mercury, - | grs. iij |
| | Arsenite of sodium, - - | grs. iij |
| | Sulphate of strychnine, - | grs. jss |
| | Carbonate of potass, | |
| | Sulphate of iron, . - - aa | grs. xiv. |

Make into sixty pills and give one after each meal.—*Clinique.—Southern Clinic.*

---

Fermentation of milk occurs only in consequence of the introduction into it of micro-organisms. If the milk be received by a sterilized tube into a sterilized receptacle directly from the udder of the cow, it will not ferment nor become acid, though kept indefinitely.—*Ex.*

